# Impact of hepatitis B virus surface antigen mutations in the major hydrophilic region on antigen detectability

**DOI:** 10.3389/fcimb.2026.1831437

**Published:** 2026-05-22

**Authors:** Chengshan He, Xuemei Gu, Yang Liu, Haitao Shen, Zhouhong Xiang, Zheng Xu

**Affiliations:** 1 Department of Laboratory Medicine, Seventh People’s Hospital of Shanghai University of Traditional Chinese Medicine, Shanghai, China; 2 Department of Clinical Laboratory, Shanghai Eighth People’s Hospital, Shanghai, China

**Keywords:** detectability, gene mutation, hepatitis B surface antigen, hepatitis b virus, major hydrophilic region

## Abstract

**Objective:**

This study aims to investigate the impact of mutations within the major hydrophilic region (MHR) of the hepatitis B virus (HBV) S gene on the expression, secretion, and antigenicity of hepatitis B surface antigen (HBsAg), and evaluate the performance of commonly used commercial HBsAg detection systems in identifying these variants.

**Methods:**

Seventeen high-frequency point mutations within the major hydrophilic region (MHR) of the HBV S gene were previously identified in individuals with occult HBV infection. These mutations were introduced into wild-type HBV sequences using site-directed mutagenesis to construct recombinant eukaryotic expression plasmids. Following sequence confirmation, the plasmids were transfected into Huh-7 cells. At 72 hours post-transfection, both culture supernatants and cell lysates were harvested. HBsAg levels were quantified using five commercial detection systems: Roche, Abbott, Autobio, CHIVD, and KHB.

**Results:**

Compared with the wild-type strain, T126A (B) and M133I (B) variants presented significantly increased HBsAg levels (P<0.05). Conversely, HBsAg expression was significantly reduced in K122E (B), C124R (B/C), M133T (B), C138R (B), T143S (B), D144G (B), and L162R (C) variants relative to the wild-type (P<0.05). The L162R (C) variant produced no detectable HBsAg in either lysates or supernatants. The C124R (B/C) variant showed higher reactivity on the Abbott and KHB systems, whereas other systems failed to detect it effectively. In contrast, the C138R (B) and M133T (B) variants were detectable in lysates but undetectable in supernatants, suggesting impaired secretion. Among the evaluated systems, Abbott and KHB systems demonstrated comparatively higher sensitivity for variant detection.

**Conclusion:**

Of the 17 MHR variants examined, only T126A (B) and M133I (B) enhanced HBsAg detectability. The remaining mutations impaired detection due to decreased expression, secretion defects, or altered antigenicity. Specifically, L162R (C) likely disrupts expression or antigenicity, C138R (B) and M133T (B) impair secretion, and C124R (B/C) alters antigenicity. Most other variants exhibited reduced detectability across systems. Continued refinement of immunoassays is needed to enhance the identification of HBsAg variants in clinical settings.

## 1 Introduction

Hepatitis B virus (HBV) infection remains a major global public health concern, with approximately 300 million individuals chronically infected worldwide (Li et al., 2020). China bears the highest burden, with an estimated 43.3 million hepatitis B surface antigen (HBsAg) carriers (Liu et al., 2023). Given the large population affected, accurate diagnosis is essential. HBsAg and HBV DNA are important serological and virological markers for the diagnosis of HBV infection. However, because nucleic acid testing requires relatively advanced laboratory infrastructure and technical support, it has not yet been fully implemented in primary healthcare institutions. Therefore, HBsAg screening remains particularly important for the diagnosis and clinical management of HBV infection, especially in primary care settings. A distinct clinical entity, known as occult HBV infection (OBI), is characterized by undetectable HBsAg in peripheral blood despite the presence of low-level HBV DNA (typically <200 IU/mL) or covalently closed circular DNA (cccDNA) in liver tissue (Raimondo et al., 2008). Failure to detect OBI can lead to misdiagnosis and delayed treatment, posing risks to blood transfusion safety and increasing the likelihood of HBV transmission through blood products. Furthermore, studies have indicated that OBI contributes to the progression of liver disease and represents a significant risk factor for hepatocellular carcinoma (HCC) development (Mak et al., 2020; Saitta et al., 2022). Several mechanisms have been proposed to explain the pathogenesis of OBI: (1) mutations in the major hydrophilic region (MHR) of the HBV S gene, particularly within the “α” determinant cluster (amino acids 124-147), which alter the antigenicity and immunogenicity of HBsAg, reducing recognition by capture or detection antibodies (Makvandi, 2016; Liu et al., 2023); (2) diminished HBsAg synthesis due to mutations in the PreS/S region and the overlapping polymerase gene, co-infection with viruses such as HIV or HCV, or impaired HBV replication capacity (Meschi et al., 2024; Sadeghi et al., 2017); (3) intracellular retention of HBsAg caused by regulatory mutations in the S gene, preventing its secretion into extracellular compartments and leading to undetectable circulating levels (Kuhns et al., 2021; He et al., 2023); and (4) limited sensitivity of current diagnostic systems to detect trace or antigenically modified forms of HBsAg. A previous study (He et al., 2024) identified 17 recurrent mutations: K122E (genotype B/C), C124R (B/C), T126A (B), I126T (C), T131A/S (B), M133I/L/T (B), C138R (B), T143S (B), D144G (B), C147R/Y (B), and L162R (C) in the MHR region of the S gene among patients with OBI. Mutation sites L162R (C) and K122E(B/C) found in this study have not been reported in previous literature. These mutations were hypothesized to be responsible for the failure of HBsAg detection and the emergence of the OBI phenotype. To investigate the mechanism by which these mutations affect HBsAg detection, site-directed mutagenesis was performed to introduce the 17 high-frequency mutations into a wild-type HBV genome. Recombinant constructs were cloned into eukaryotic expression vectors and transfected into Huh-7 cells. Supernatants and cell lysates containing mutant HBsAg proteins were collected for analysis. Five widely used HBsAg detection systems, including Cobas e602 (Roche, Switzerland), ARCHITECT i2000 (Abbott, USA), Lumo A2000Plus (Autobio, Zhengzhou, China), LiCA^®^ 500 (CHIVD, Beijing, China), and Polaris i2400 (KHB, Shanghai, China), were used to assess detection performance. The study aimed to evaluate the detection efficacy of these systems for mutant HBsAg proteins and to explore the impact of MHR mutations on HBsAg expression, secretion, and antigenicity.

## 2 Materials and methods

### 2.1 Common mutations in the HBV S gene associated with OBI

Based on previous findings (He et al., 2024), 17 common mutations within the MHR of the HBV S gene were selected from serum samples of OBI cases, characterized by HBsAg negativity and HBV DNA positivity. The selected mutations included K122E (B/C), C124R (B/C), T126A (B), I126T (C), T131A/S (B), M133I/L/T (B), C138R (B), T143S (B), D144G (B), C147R/Y (B), and L162R (C). The corresponding HBV subtypes used for plasmid construction were AB602818 (genotype B) and AB014381 (genotype C). The study protocol was approved by the Medical Ethics Committee of our hospital (No. 2024-7th-HIRB-068).

### 2.2 Primer design

The complete sequences of AB602818 (genotype B) and AB014381 (genotype C) were retrieved from the NCBI database (https://blast.ncbi.nlm.nih.gov/Blast.cgi) and used as wild-type HBV DNA templates. Based on these reference sequences, primers for site-directed mutagenesis were designed using Primer5 software to introduce 17 amino acid substitutions. All primers were synthesized by Huajin Biotech Co., Ltd. (Shanghai, China). The sequences and details are listed in **Table 1**.

**Table 1:** Primers used for site-directed mutagenesis.

Primer	Orientation	Primer sequence (5′-3′)
P1	Forward	TTGGTACCGAGCTCGGCCACCATGGAGAACATCGCATCAGGACTCC
	Reverse	CTGGATATCTGCAGAATTTTAAATGTATACCCAAAGACAAAAGAAAATT
K122E (B)	Forward	GCACAACTCCTGCTCAAGGAACCTCTATGT
	Reverse	TGAGCAGGAGTTGTGCAGGTCTCGCATGG
K122E (C)	Forward	ACCATGCGAGACCTGCACGATTCC
	Reverse	TGAGCAGGAATCGTGCAGGTC
C124R (B)	Forward	CAAGACCCGCACAACTCCTGCTCAAGG
	Reverse	AGTTGTGCGGGTCTTGCATGGTCCC
C124R (C)	Forward	GGACCATGCAAGACCCGCACGATTCC
	Reverse	CCTTGAGCAGGAATCGTGCGGGTC
T126A (B)	Forward	CACAGCTCCTGCTCAAGGAACCTC
	Reverse	TTGAGCAGGAGCTGTGCAGGTCTTG
I126T (C)	Forward	CCTGCACAATTCCTGCTCAAGGAACC
	Reverse	GCAGGAATTGTGCAGGTCTTGCAT
T131A (B)	Forward	TTTCCCTCATGTTGCTGTACAAAACCTACG
	Reverse	CAGCAACATGAGGGAAACATAGAGGCTCCTTGA
T131S (B)	Forward	GTTTCCCTCATGTTGCTGTACAAAACCTAC
	Reverse	GCAACATGAGGGAAACATAGAGGATCCTTGAG
M133I (B)	Forward	CTCTATATTTCCCTCATGTTGCTGTACAAAACCTACG
	Reverse	ACATGAGGGAAATATAGAGGTTCCTTGAGCAG
M133L (B)	Forward	CCTCATGTTGCTGTACAAAACCTACGG
	Reverse	TGTACAGCAACATGAGGGAAACAGAGAGGTT
M133T (B)	Forward	ACCTCTACGTTTCCCTCATGTTGCTGTACA
	Reverse	TGAGGGAAACGTAGAGGTTCCTTGAGCA
C138R (B)	Forward	CAAAACCTACGGATGGAAACTGCACC
	Reverse	TCCATCCGTAGGTTTTGTACAGCGACATGA
T143S (B)	Forward	ACCTTCGGATGGAAACTGCACCTG
	Reverse	GTTTCCATCCGAAGGTTTTGTACAGCAAC
D144G (B)	Forward	ACTGCACCTGTATTCCCATCCCA
	Reverse	GGAATACAGGTGCAGTTTCCACCCGTAGGT
C147R (B)	Forward	TGGAAACCGCACCTGTATTCCCATCCCATCAT
	Reverse	CAGGTGCGGTTTCCATCCGTAGGTTTTG
C147Y (B)	Forward	TGGAAACTACACCTGTATTCCCATCCCATCA
	Reverse	CAGGTGTAGTTTCCATCCGTAGGTTTTG
L162R (C)	Forward	GCTTTCGCAAGATTCCGATGGGAGTGG
	Reverse	GGACTGAGGCCCACTCCCATCGGAATC

### 2.3 Construction of eukaryotic expression vectors with HBV S gene mutations

Serum samples with positive HBsAg, hepatitis B e antigen (HBeAg), and hepatitis B core antibody (anti-HBc), as well as HBV DNA levels exceeding 1×10^6^ IU/mL, were selected. HBV DNA was extracted using a nucleic acid magnetic bead isolation kit (PerkinElmer, USA). The concentration of extracted DNA (ng/mL) was measured using a UV spectrophotometer (NanoDrop, Thermo Fisher Scientific, USA). PCR amplification was performed using P1 primers (reaction conditions described in section 2.4). The resulting PCR products were sequenced (Azenta Biotechnology Co., Ltd., Tianjin, China), and the sequences were aligned with reference strains from the NCBI database to determine genotypes (www.ncbi.nlm.nih.gov/projects/genotyping/). Samples confirmed as AB602818 (genotype B) and AB014381 (genotype C) with no amino acid substitutions in the S gene region were selected as wild-type HBV B/C genotype templates for this study.

### 2.4 Construction of eukaryotic expression vectors with site-directed mutations

Eukaryotic expression vectors were constructed using the pcDNA3.1 backbone (Thermo Scientific, USA). Based on the wild-type HBV template, site-directed mutagenesis was performed to introduce specific amino acid substitutions at specific sites. The procedure was carried out as follows: (1) the upstream fragment of the HBV S gene containing the mutation site was amplified using the forward P1 primer and the reverse mutation-specific primer; (2) the downstream fragment was amplified using the reverse P1 primer and the forward mutation-specific primer; (3) full-length wild-type sequences were amplified using the P1 primer pair as the control; (4) the pcDNA3.1 vector was linearized using Fast Digest EcoR I and BamH I restriction endonucleases (Thermo Scientific, USA); (5) the two PCR-amplified fragments (upstream and downstream) were ligated into the linearized vector using a seamless cloning kit (Sangon Biotech, Shanghai, China). The recombinant plasmids were transformed into competent E. coli DH5α cells (New Cell & Molecular Biotech, Suzhou, China). Positive colonies were screened and verified by Sanger sequencing (Azenta Biotech, Tianjin, China). Sequences showing correct site-directed mutations were designated as recombinant mutant expression plasmids. The primer sequences used for PCR are listed in **Table 1**. PCR amplification was conducted using an ABI Veriti Thermal Cycler (ABI, USA). Each 50 µL reaction mixture contained 20 ng of DNA template, 25 µL of Takara Premix Taq polymerase mixture (Takara Biomedical, Beijing, China), 2 µL of primer mix, and DEPC-treated water to volume. The PCR conditions were as follows: initial denaturation at 98 °C for 30 s; 30 cycles of denaturation at 98 °C for 10 s, annealing at 55 °C for 30 s, and extension at 72 °C for 65 s.

### 2.5 Expression of mutant HBsAg

Recombinant plasmids verified by sequencing in section: Construction of eukaryotic expression vectors with site-directed mutations (including wild-type and mutant constructs) were inoculated into LB broth and cultured overnight in a shaker. Plasmid DNA was extracted using an endotoxin-free plasmid mini prep kit (Tiangen Biotech, Beijing, China). Huh-7 cells (Wanwu Biotechnology, Hefei, China) were seeded in six-well plates 24 hours prior to transfection to achieve 70%-80% confluency at the time of transfection. Transfection was performed using the Lipofectamine™ 3000 reagent (Thermo Scientific, USA) according to the manufacturer’s instructions. Cells were incubated at 37 °C in a humidified CO_2_ incubator following transfection. After 72 hours, cell culture supernatants were collected. Then, 300 μL of RIPA lysis buffer (New Cell & Molecular Biotech, Suzhou, China) was added to each well, and cells were lysed on ice for 40 minutes. Lysates were clarified by centrifugation to obtain the soluble fraction. Both the collected supernatants and cell lysates were stored at -80 °C for subsequent analysis.

### 2.6 Detection of mutant HBsAg

Five clinically validated HBsAg detection systems were employed: Cobas e602 (Roche), ARCHITECT i2000 (Abbott), Lumo A2000 (Autobio), LiCA^®^ 500 (CHIVD), and Polaris i2400 (KHB). All assays were conducted using system-specific reagent kits and calibrators. Internal quality control materials were obtained from the Shanghai Clinical Testing Center (China). Protein concentrations of the cell lysates were measured and normalized to 0.25 mg/mL using a BCA protein assay kit (Sangon Biotech, Shanghai, China). Each sample was tested in duplicate, and the mean value was used for analysis. Supernatants were directly tested without dilution, and the mean value of two replicate measurements was recorded.

### 2.7 Statistical analysis

We used SPSS 19.0 for data processing and statistical analysis. Comparisons between the two groups were performed using Student’s t-test, with P<0.05 (two sided) considered statistically significant.

## 3 Results

### 3.1 Sequencing verification of recombinant eukaryotic expression vectors with point mutations

**Figure 1:**
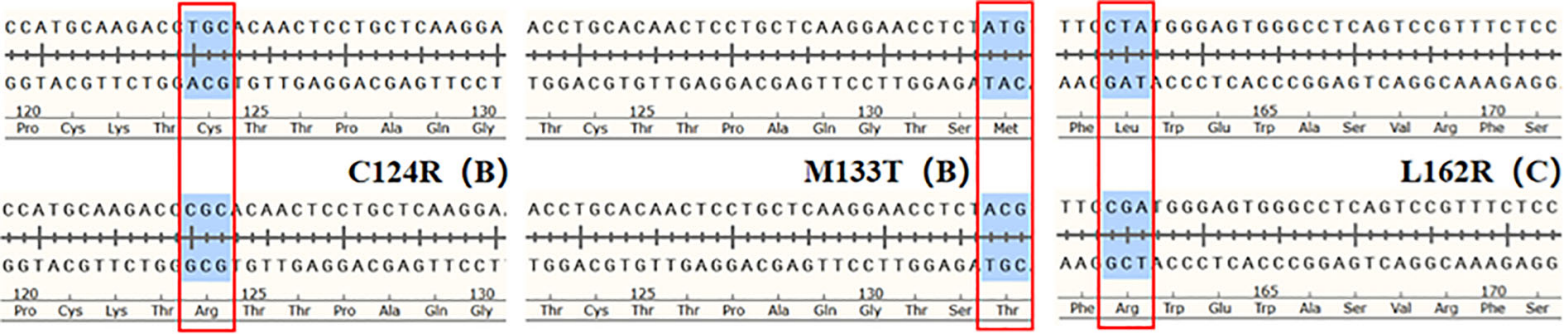
Graphical result of recombinant eukaryotic expression vectors for the C124R (B), M133T (B), and L162R (C) mutations.

Following site-directed mutagenesis, PCR fragments carrying point mutations, including K122E (B/C), C124R (B/C), T126A (B), I126T (C), T131A/S (B), M133I/L/T (B), C138R (B), T143S (B), D144G (B), C147R/Y (B), and L162R (C), were successfully inserted into the eukaryotic expression vector pcDNA3.1. Sanger sequencing confirmed the accuracy of all recombinant plasmids by alignment with the reference sequences. Representative sequencing alignments are shown in **Figure 1**.

### 3.2 Detection of intracellular mutant HBsAg in cell lysates by five HBsAg systems

Five clinically used HBsAg detection systems (Roche, Abbott, Autobio, CHIVD, and KHB), along with their matched reagents and calibrators, were used to evaluate the detection performance for intracellular mutant HBsAg. Huh-7 cells were transfected with plasmids encoding each of the 17 HBV S gene mutations. At 72 hours post-transfection, HBsAg in cell lysates was measured to assess the recognition ability of each detection system for intracellular variant HBsAg. All five systems demonstrated 100% concordance in identifying HBsAg-negative signals for the negative control (pcDNA3.1) and HBsAg-positive signals for the wild-type HBV control. Among the five HBsAg systems, T126A (B) and M133I (B) variants exhibited markedly higher HBsAg levels than the wild-type strain (P<0.05). Conversely, HBsAg expression was significantly decreased in K122E (B), C124R (B/C), M133T (B), C138R (B), T143S (B), D144G (B), and L162R (C) variants relative to the wild-type (P<0.05). Notably, none of the five systems detected HBsAg in lysates from cells transfected with the L162R (C) mutant. Additionally, the Roche, Autobio, and CHIVD systems failed to detect HBsAg from the C124R (B/C) mutant, and the Autobio system showed no reactivity to the C138R (B) mutant. For the remaining mutations, such as K122E (B/C), T126A (B), I126T (C), T131A/S (B), M133I/L/T (B), T143S (B), D144G (B), and C147R/Y (B), all five systems displayed varying degrees of reactivity. Detailed detection performance for each mutant is presented in **Table 2**.

**Table 2:** Detection performance of five HBsAg systems for cell lysates (intracellular) and supernatants (extracellular) mutant HBsAg.

Mutation site	HBsAg detection systems (IU/mL)									
	Cell lysate (Intracellular)					Supernatant (Extracellular)				
	Roche	Abbott	Autobio	CHIVD	KHB	Roche	Abbott	Autobio	CHIVD	KHB
pc DNA 3.1	*	*	0.01	0.03	0.02	*	*	0.01	0.03	0.01
K122E (B)	0.30	1.44	0.95	0.63	0.76	0.11	0.36	0.49	0.28	0.42
K122E (C)	3.40	6.06	3.98	4.05	7.37	3.61	7.23	4.78	6.14	10.72
C124R (B)	0.04	0.34	0.01	0.04	1.80	*	0.25	0.01	0.03	1.09
C124R (C)	0.03	0.57	0.03	0.04	3.04	0.04	1.32	0.22	0.03	5.03
T126A (B)	9.08	9.13	4.86	7.39	13.19	4.87	7.94	3.65	4.59	8.10
I126T (C)	5.60	4.91	5.37	6.48	10.54	1.11	0.50	1.02	0.80	1.14
T131A (B)	1.85	2.74	1.44	1.34	1.53	0.35	0.37	0.30	0.18	0.19
T131S (B)	2.94	3.13	1.25	1.98	1.88	1.89	2.75	1.42	1.76	1.98
M133I (B)	3.65	4.12	1.65	2.21	3.87	3.50	4.86	2.05	3.07	4.98
M133L (B)	1.86	1.92	1.02	0.69	1.51	1.64	2.66	1.11	1.42	2.13
M133T (B)	0.96	0.99	0.74	0.74	0.69	0.04	0.02	0.01	0.04	0.03
C138R (B)	0.68	0.08	0.01	0.71	1.95	0.04	*	0.01	0.04	0.03
T143S (B)	1.50	1.05	0.85	0.90	1.25	0.76	0.64	0.61	0.60	0.48
D144G (B)	1.46	1.00	0.84	0.58	1.60	0.27	0.20	0.21	0.32	0.35
C147R (B)	4.40	1.60	1.13	1.74	4.53	1.43	0.44	0.71	0.57	1.81
C147Y (B)	3.87	2.07	0.37	0.95	3.42	1.55	1.13	0.22	0.56	2.27
L162R (C)	0.03	0.01	0.01	0.03	0.01	*	0.01	0.01	0.02	0.01
Wide-type B	2.31	2.26	1.38	1.23	1.98	2.10	2.95	1.57	1.86	2.67
Wide-type C	9.48	6.35	4.48	13.61	11.71	13.07	16.19	10.84	24.31	28.63

The detection threshold for all five systems (Roche, Abbott, Autobio, CHIVD, and KHB) was set at ≥ 0.05 IU/mL. Values ≥ 0.05 IU/mL were considered reactive, and values < 0.05 IU/mL were considered non-reactive; *No valid concentration was detected.

### 3.3 Detection of secreted mutant HBsAg in cell culture supernatants by five HBsAg systems

The same five HBsAg detection systems and their corresponding reagents were used to evaluate the recognition of secreted mutant HBsAg in culture supernatants. Huh-7 cells were transfected with plasmids encoding the 17 HBV S gene mutations, and culture supernatants were collected at 72 hours post-transfection. The ability of each system to detect extracellular HBsAg variants was assessed. All five systems correctly identified HBsAg-negative signals from the pcDNA3.1 negative control and HBsAg-positive signals from the wild-type HBV control, achieving 100% concordance. Among the five HBsAg systems, T126A (B) and M133T (B) variants exhibited markedly higher HBsAg levels than the wild-type strain (P < 0.05). Conversely, HBsAg expression was significantly decreased in K122E (B/C), C124R (B/C), T131A (B), M133T (B), C138R (B), T143S (B), D144G (B), C147R/Y (B), I126T (C), and L162R (C) variants relative to the wild-type (P<0.05). None of the systems detected secreted HBsAg in supernatants from cells transfected with the M133T (B), C138R (B), and L162R (C) variants. Additionally, both the Roche and CHIVD systems failed to detect the C124R (B/C) variant, while the Autobio system did not detect the C124R (B) mutation. For other mutations K122E (B/C), T126A (B), I126T (C), T131A/S (B), M133I/L (B), T143S (B), D144G (B), and C147R/Y (B) all five systems exhibited varying levels of detection. The comparative detection results for intracellular and extracellular HBsAg are summarized in **Table 2**.

### 3.4 Comparative detection performance of five HBsAg systems for mutant HBsAg

To compare the detection performance of the five systems for mutant HBsAg, both cell lysates and corresponding supernatants from Huh-7 cells transfected with 17 mutant expression plasmids were tested, resulting in a total of 34 samples. The positive detection rates for mutant HBsAg were as follows: Roche, 76.47% (26/34); Abbott, 88.24% (30/34); Autobio, 76.47% (26/34); CHIVD, 76.47% (26/34); and KHB, 88.24% (30/34). A detailed comparison of detection performance across the 17 individual mutations is presented in **Table 3**. Among the systems evaluated, Abbott and KHB demonstrated superior sensitivity in detecting mutant HBsAg, outperforming the other three systems.

**Table 3:** Comparative detection performance of five HBsAg systems for 17 HBsAg variants.

Mutation site	Non-reactivity HBsAg detection systems	
	Cell lysate (Intracellular)	Supernatant (Extracellular)
K122E (B)	None	None
K122E (C)	None	None
C124R (B)	Roche, Autobio, CHIVD	Roche, Autobio, CHIVD
C124R (C)	Roche, Autobio, CHIVD	Roche, CHIVD
T126A (B)	None	None
I126T (C)	None	None
T131A (B)	None	None
T131S (B)	None	None
M133I (B)	None	None
M133L (B)	None	None
M133T (B)	None	Roche, Abbott, Autobio, CHIVD, KHB
C138R (B)	Autobio	Roche, Abbott, Autobio, CHIVD, KHB
T143S (B)	None	None
D144G (B)	None	None
C147R (B)	None	None
C147Y (B)	None	None
L162R (C)	Roche, Abbott, Autobio, CHIVD, KHB	Roche, Abbott, Autobio, CHIVD, KHB

### 3.5 Comparison of mutant and wild-type HBsAg concentrations

**Figure 2:**
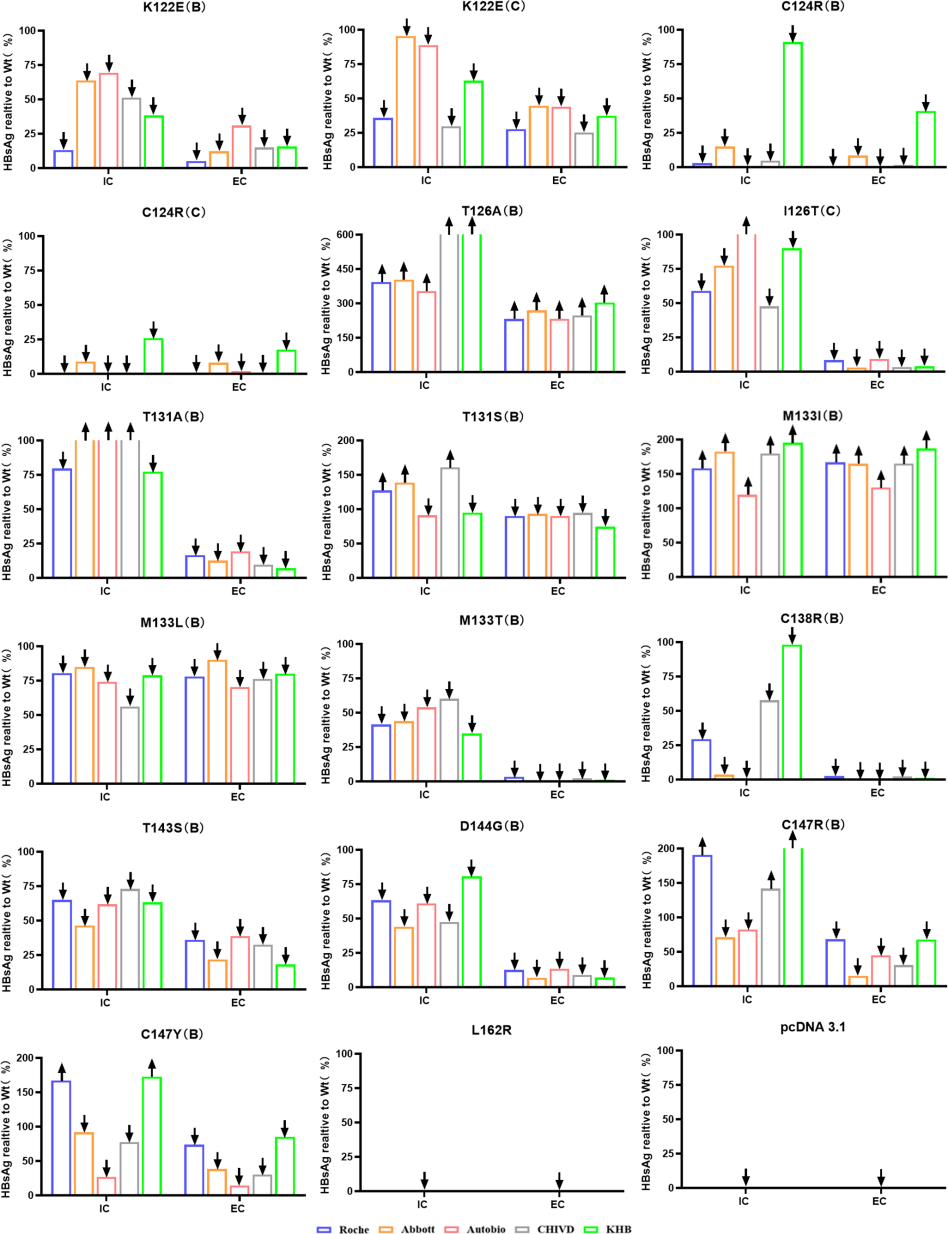
Comparative concentrations of recombinant mutant and wild-type HBsAg across different detection systems. Cell lysate (Intracellular, IC), Supernatant (Extracellular, EC); The open top of the column chart indicates that the corresponding value (%) exceeds the upper limit of the vertical axis (HBsAg realtive to Wt).

To assess the impact of mutations on HBsAg expression, the concentration of mutant HBsAg in both cell lysates and supernatants was compared to the corresponding wild-type HBsAg concentration, expressed as the mutant/wild-type ratio. This ratio was used to evaluate differences in HBsAg expression and secretion between mutant and wild-type forms. In cell lysates, the T126A (B) and M133I (B) mutations resulted in higher HBsAg concentrations than wild-type across all five systems (P<0.05). Specifically, T126A (B) showed a 3.5- to 6.7-fold increase, while M133I (B) showed a 1.2- to 2.0-fold increase. In contrast, mutations C124R (B/C) and L162R (C) led to markedly reduced HBsAg expression. For example, C124R (B) yielded Mt/Wt ratios ranging from 0.72% to 15.04% (90.91% in KHB), and C124R (C) yielded ratios from 0.29% to 8.98% (25.96% in KHB). L162R (C) produced undetectable HBsAg levels in all systems. Some systems showed elevated HBsAg concentrations for mutations such as I126T (C), T131A/S (B), M133I (B), and C147R/Y (B), though most other mutations resulted in lower concentrations compared to wild-type. In culture supernatants, only T126A (B) and M133I (B) mutants showed higher HBsAg concentrations than wild-type (P<0.05), with increases ranging from 1.3- to 3.0-fold. All other mutations exhibited decreased expression relative to wild-type. Notably, mutations such as C124R (B/C), I126T (C), C138R (B), M133T (B), and L162R (C) had mutant/wild-type ratios averaging only 0%-10%, with L162R (C) remaining undetectable across all systems. For all mutations, mutant/wild-type ratios in supernatants were consistently and substantially lower than those in cell lysates, suggesting impaired secretion of mutant HBsAg proteins. A summary of the mutant/wild-type concentration comparisons across detection platforms is shown in **Figure 2**.

## 4 Discussion

During HBV infection, both complete virions and an overwhelming excess of subviral particles (SVPs) are secreted from hepatocytes into the peripheral circulation. SVPs, which outnumber virions by several orders of magnitude, are 20-nm spherical or filamentous structures composed solely of lipids and viral envelope proteins. Sharing the same antigenicity as virions, SVPs constitute the principal source of hepatitis B surface antigen (HBsAg) in the bloodstream. (Heermann et al., 1984). As a key serological marker, HBsAg is widely used in clinical practice for blood donor screening, diagnosis of HBV, monitoring of chronic hepatitis B, and evaluation of antiviral therapy efficacy.To investigate the effects of MHR mutations in the HBV S gene on HBsAg expression, secretion, and antigenicity, as well as their impact on the performance of commonly used clinical HBsAg detection systems, 17 site-directed mutants were constructed, including K122E (B/C), C124R (B/C), T126A (B), I126T (C), T131A/S (B), M133I/L/T (B), C138R (B), T143S (B), D144G (B), C147R/Y (B), and L162R (C). The mutant constructs were transfected into Huh-7 cells, and both cell lysates and culture supernatants were collected for analysis. Quantitative analysis of HBsAg was conducted using five widely adopted commercial detection systems. Among all variants tested, the L162R (C) mutation resulted in undetectable HBsAg levels in both lysates and supernatants across all systems, indicating a complete loss of assay reactivity. Two potential mechanisms may underlie this phenomenon: first, the mutation may severely impair HBV gene expression and replication, leading to reduced HBsAg production; second, the mutation may alter the antigenic structure of HBsAg such that capture or detection antibodies in conventional immunoassays fail to recognize the epitope. Nevertheless, both situations ultimately appear as negative HBsAg results in immunological assays.

Previous studies have suggested that the s162 is located in the third transmembrane domain (TMD3, amino acids 160-193), which is currently less studied (Jiang et al., 2021, 2022). Currently, the topological structure of TMD3 has not yet been fully determined. However, we do know that it contains a “α” helical structure. Therefore, in this study TMD3 and MHR are found to share overlapping regions from mino acids 160 to 169. TMD is predominantly composed of hydrophobic amino acids. The substitution of a hydrophobic leucine with a positively charged arginine may disrupt the hydrophobic character of TMD3. Additionally, the introduction of a charged residue may interfere with protein folding and membrane insertion within the endoplasmic reticulum, potentially resulting in intracellular retention and accelerated degradation of HBsAg (Zhang et al., 2019). Previous studies have demonstrated that mutations within the second transmembrane domain (TMD2, amino acids 80-98), including C85R, L87R, L88R, and C90R, impair both expression and secretion of HBsAg (Zhang et al., 2019). Therefore, the non-reactivity of the L162R (C) variant may be attributable to similar mechanisms. Given the limitations of immunoassays in distinguishing between reduced antigen expression level and altered antigenicity, both mechanisms may contribute to the observed failure of HBsAg detection. Future studies will employ mass spectrometry-based proteomic approaches to confirm whether the L162R (C) mutation still permits intracellular expression of variant HBsAg protein, thereby clarifying the underlying cause of the assay non-reactivity.

For the C124R (B/C) mutation, the KHB system demonstrated superior detection performance compared to the other systems, particularly for the C124R (B) variant, where the mutant/wild-type ratio reached approximately 70%. In contrast, the remaining detection systems showed poor reactivity to both C124R (B) and C124R (C) in cell lysates and supernatants. It is widely recognized that the “α” determinant region (amino acids 124-147) is the immunodominant domain of HBsAg, in which two loop structures, formed between C124-C137 and C139-C147, are essential for maintaining the protein’s conformational integrity and antigenicity (Huang et al., 2017; Zhang et al., 2018). The cysteine residue at position s124 is located at the beginning of the first loop and forms a disulfide bond with the cysteine at position s147. Mutations at these conserved sites can disrupt the conformational epitope, directly altering HBsAg antigenicity and reducing the binding affinity of capture or detection antibodies in commercial assays. This structural alteration likely contributes to the diminished detectability of C124R (B/C) HBsAg in both lysates and supernatants across most systems (Carman, 1998; Lee et al., 1997). Consistent with our findings, previous studies have reported that the C124R mutation leads to reduced antigenicity of HBsAg and impaired secretion of viral particles in both Huh-7 cells and mouse models (Huang et al., 2012).

The T126A (B) and M133I (B) mutations resulted in significantly higher concentrations of HBsAg compared to the wild-type (P<0.05). Specifically, T126A (B) showed a 3.5- to 6.7-fold increase in cell lysates and a 2.3- to 3.0-fold increase in culture supernatants. M133I (B) showed a 1.3- to 2.0-fold increase in lysates and a 1.2- to 1.9-fold increase in supernatants. These findings indicate that not all mutations within the “α” determinant necessarily weaken HBsAg detectability. Certain mutations within the loop region may even enhance the antigenicity of HBsAg. Previous studies have reported that the s126 residue may exist in three amino acid forms: s126I, s126T, and s126A, all of which exhibit similar binding affinities to anti-HBs antibodies (HBsAb) (Qiu et al., 2008). These findings suggest that the T126A mutation is unlikely to impair the detectability of HBsAg. Furthermore, Hyon Suk Kim et al (Kim et al., 2018) identified the T126A and M133I mutations in HBV genomes derived from HBsAg-positive samples detected using the Roche Elecsys® HBsAg II assay, supporting the notion that these mutations do not reduce assay reactivity and may even enhance antibody recognition.

The M133T (B) and C138R (B) mutations exhibited partial reactivity in cell lysates across all five detection systems, with mean mutant/wild-type ratios of 46.80% and 37.98%, respectively, indicating a moderate reduction in HBsAg detectability compared to the wild-type (P<0.05). However, in culture supernatants, neither mutation produced detectable levels of HBsAg. Given that intracellular HBsAg was measurable while extracellular HBsAg remained undetectable, these mutations may impair HBsAg secretion, leading to false-negative results in peripheral blood. Previous studies have shown that the M133T mutation can create a novel N-glycosylation site when accompanied by T131N, forming the T131N/M133T motif. This additional glycosylation site may interfere with B-cell epitope recognition and disrupt the antigenicity of HBsAg. In Huh-7 cells transfected with the T131N/M133T mutant, the antigenic and immunogenic properties of HBsAg were substantially impaired. Mice immunized with the pHBsAg-T131N/M133T plasmid exhibited a significantly reduced HBsAb response, although HBV replication *in vivo* remained unaffected (Kang et al., 2018), suggesting that the mutation influences antigen presentation rather than expression levels. In our current study, the T131N substitution was not observed in conjunction with the M133T mutation. Nonetheless, the reduction in antigenicity of the M133T variant was evident. For C138R (B), detailed characterization remains limited in the literature. Based on our experimental findings, we propose that both M133T (B) and C138R (B) may contribute to OBI by impairing HBsAg secretion, rather than affecting its expression.

Furthermore, for mutations such as K122E (B/C), I126T (C), T131A/S (B), M133L (B), T143S (B), D144G (B), and C147R/Y (B), the mutant/wild-type ratios of HBsAg concentration in culture supernatants were consistently below 1, indicating varying degrees of reduced detectability. In contrast, mutations including I126T (C) (detected by Autobio), T131S (B) (detected by Roche, Abbott, and CHIVD), C147R (B) (detected by Roche, CHIVD, and KHB), and C147Y (B) (detected by Roche and KHB) exhibited mutant/wild-type ratios greater than 1 in cell lysates. These findings suggest that while intracellular expression of these HBsAg variants may be maintained or even elevated compared to wild-type, their secretion into the extracellular space is often impaired. Collectively, the markedly lower levels of variant HBsAg in culture supernatants compared to cell lysates suggest that secretion defects are a common consequence of mutations within the MHR of the HBV S gene. Most amino acid substitutions in this region appear to disrupt proper protein processing or trafficking, thereby reducing the detectability of HBsAg by standard immunoassays. Among the five commercial HBsAg detection systems evaluated, the Abbott and KHB systems demonstrated relatively higher sensitivity for mutant HBsAg. In contrast, the other systems showed reduced ability to detect certain variants, highlighting the need for further refinement. Enhancing assay performance could involve incorporating broadly reactive antibody pairs targeting conserved and escape-prone epitopes, increasing antibody coating densities, or adding signal-amplifying agents to improve detection sensitivity.

We attempted to use Western blotting (WB) to further validate the performance of mutant HBsAg observed by Electrochemiluminescence immunoassay (ECLIA) and chemiluminescence immunoassay (CLIA). Unfortunately, the results were not as expected. One possible explanation is the difference in analytical sensitivity between the two methods. ECLIA and CLIA can detect HBsAg at the IU/mL level. Based on the conversion of the standard measurement unit from ng/mL to IU/mL, with an approximate ratio of 1:2 (World Health Organization, 2004), even the highest HBsAg level detected in the wild-type virus was only about 14 ng/mL, whereas most measured values were below 5 IU/mL, equivalent to approximately 0.025-2.5 ng/mL. This extremely low concentration of HBsAg may limit the application of WB assay. In addition, the HBsAg monoclonal antibodies selected for our experiments exhibit certain limitations in variant antigen recognition, as compared with polyclonal antibody preparations.

This study demonstrated that mutations within the MHR of the HBV S gene can significantly affect the detectability of HBsAg using commercial immunoassays. Among the 17 variants tested, only T126A (B) and M133I (B) mutations were found to enhance HBsAg detection, while the majority of other mutations led to decreased detection performance. These impairments may result from reduced HBsAg expression, impaired secretion, altered antigenicity, or suboptimal assay sensitivity. Specifically, the L162R (C) mutation appears to affect either the expression or antigenic profile of HBsAg; the M133T (B) and C138R (B) mutations likely impair secretion; and the C124R (B/C) mutation alters antigenicity. Among the five detection systems evaluated, the Abbott and KHB systems showed relatively superior sensitivity to HBsAg variants. Nonetheless, the ability of current assays to reliably detect diverse HBsAg mutants remains limited, highlighting the need for further optimization in diagnostic reagent design and validation.This study also has several limitations. First, the analysis was restricted to mutations within the MHR region of the HBV S gene. Future studies with larger sample sizes and broader mutation coverage, including full-length HBV genome sequencing, are warranted. Second, the study focused primarily on single-point mutations, while naturally occurring HBV strains often harbor complex or combinatorial mutations that may have synergistic effects on HBsAg properties. Third, the study is based on an *in vitro* overexpression system, which may not fully recapitulate physiological viral infection or antigen presentation. Finally, additional investigations are needed to elucidate the exact mechanisms underlying the detection failure of the L162R (C) mutation, including studies on protein expression, intracellular trafficking, and antigen-antibody interactions.

## Data Availability

The original contributions presented in the study are included in the article/supplementary material. Further inquiries can be directed to the corresponding author.
